# Activity and Participation Characteristics of Adults with Learning Disabilities - A Systematic Review

**DOI:** 10.1371/journal.pone.0106657

**Published:** 2014-09-03

**Authors:** Kineret Sharfi, Sara Rosenblum

**Affiliations:** The Laboratory of Complex Human Activity and Participation, Department of Occupational Therapy, Haifa University, Haifa, Israel; University of Perugia, Italy

## Abstract

**Background:**

‘Learning disabilities’ (LD) refer to a wide group of neurological disorders caused by deficits in the central nervous system which influence the individual's ability to maintain-, process or convey information to others in an efficient way. A worldwide discussion about the definitions of LD continues while a conceptual framework for studying the diverse life outcomes of adults with LD is still missing.

**Objective:**

The aim was to review the literature on the *activity* and *participation* of adults with LD based on the International Classification of Functioning, Disability and Health (ICF) concepts.

**Methods:**

“PsychInfo”, “Eric” and “PubMed” were searched for relevant literature according to the guidelines of Preferred Reporting Items for Systematic Reviews and Meta-Analyses (PRISMA). After a three-stage process, 62 articles relevant for domains of activity and participation of adults with LD were included in the review.

**Results:**

Thirty-two articles focused on the domain of major life areas of education, work and employment and twelve articles focused on the domain of learning and applying knowledge. Limitations in activity and participation of the population with LD in these domains are recognized and discussed. Eighteen additional articles demonstrated that adults with LD confront difficulties in various life domains (e.g., communication, interpersonal interactions, mobility, and domestic life), however literature concerning these domains is scarce.

**Conclusions:**

The ICF can be useful for further exploration of activity and participation characteristics of adults with LD in various life domains. Such exploration is required in order to gain a wider perspective of their functional characteristics and daily needs.

## Introduction

The term “learning disabilities” (LD) refers to a wide group of neurological disorders caused by deficits in the central nervous system which influence the individual's ability to maintain, process or convey information to others in an efficient way (e.g.[1]). The discussion about the definition of LD is continuing worldwide (e.g. [2]) and consequently, the data regarding the prevalence of LD in the total population has also been argued. As far as is known, there is no formal up to date information about the prevalence of LD among adults however each year very large numbers of students with LD leave high school and begin their adult lives, facing a wide variety of challenges leading to a broad array of outcomes [3].

Common definitions of LD (e.g. [4], [5]) emphasize a specific nature of the LD and focus on the deficient academic skills [6]. Even though, previous less known definitions of LD have exhibited difficulties in activity and participation in other life domains among people with LD, for example in *work and daily functioning* [7], *organizational skills, perception, social interaction and life perspective* [8]. The new APA DSM-5 version includes a suggestion to make a diagnosis of LD based on a clinical synthesis of developmental, medical, family, and educational reports [9]. This change may reflect a current understanding of the complicated nature of the LD and the need for gathering comprehensive data on activity and participation of the person in a variety of environments. Qualitative literature also indicates that the daily picture of people with LD includes deficiencies in activity and participation in various life domains such as friendship and dating, partnerships, parenting, and general perception of quality of life (e.g. [10]).

Further complexity of the issue of LD is caused due to a high co-occurrence of the LD with other associated disorders, for example: Attention Deficit\Hyperactivity Disorder (ADD\ADHD) (e.g. [11]); Developmental Coordination Disorder (DCD) (e.g. [12]); Difficulties in social skills (e.g. [13]); Depression and anxiety disorders (e.g. [14]). Some investigators included people with specific LD such as reading, writing, spelling and arithmetic deficits together with DCD, ADHD and Specific Language Impairment (SLI) in a broad perspective termed Atypical Brain Development (ABD) (e.g. [15]). All of these disorders required unique diagnosis and intervention methods to improve daily functions [16].

The on-going discussion about the definition of LD, the broad array of outcomes, the hints regarding difficulties in activity and participation in various life domains and the high co-occurrence of LD with other associated disorders imply that a health perspective, as the International Classification of Functioning, Disability and Health (ICF) model may be useful in order to gain further understanding of the daily needs of this population and consequently to improve their quality of life.

The International Classification of Functioning, Disability and Health (ICF) developed by the World Health Organization [17], is a comprehensive health model designed to classify and assess health and health-related issues and their connection to daily activity and participation. According to the ICF model the term “activity” describes the execution of specific tasks while the term “participation” describes involvement in a range of life situations [17]. The components Activity and Participation are presented separately in the model however the list of domains for these two components is one list (see Figure S1). It is important to note, however, that although activity and participation are central concepts of the ICF model, their translation and implications to research, evaluation and intervention methods among populations with specific diagnoses are still in their primary steps [18].

Following the ICF concepts, LD can be considered as *health conditions* since the term LD is defined as “deficits which influence a person's ability” (e.g. [1]). More specifically, LD is “caused by deficits in the central nervous system (CNS)…” [1], implying that the origin of LD is in *body structures and functions*. The neurological origin of LD is widely acknowledged and is supported by scientific evidence (e.g. [11], [19]). According to LDA Canada (2002), the expressions of LD are affected by interactions between environmental demands and the strengths and needs of the afflicted person [8], meaning *contextual factors* are also related to the levels of functioning and disability as expressed in the daily *activity and participation* of adults with LD.

The purpose of this review is to describe literature regarding the daily activity and participation of adults with LD in various life domains using the ICF model concepts. It is important to note, however, that unlike most of the existing research, which attempts to specify the type of LD they present, in light of the information previously described about the intricacy of LD no specification of LD types will be conducted. All types of specific LD known in the literature will be included in this review and generally related to- as LD.

## Methods

### Search strategy

**Figure 1:**
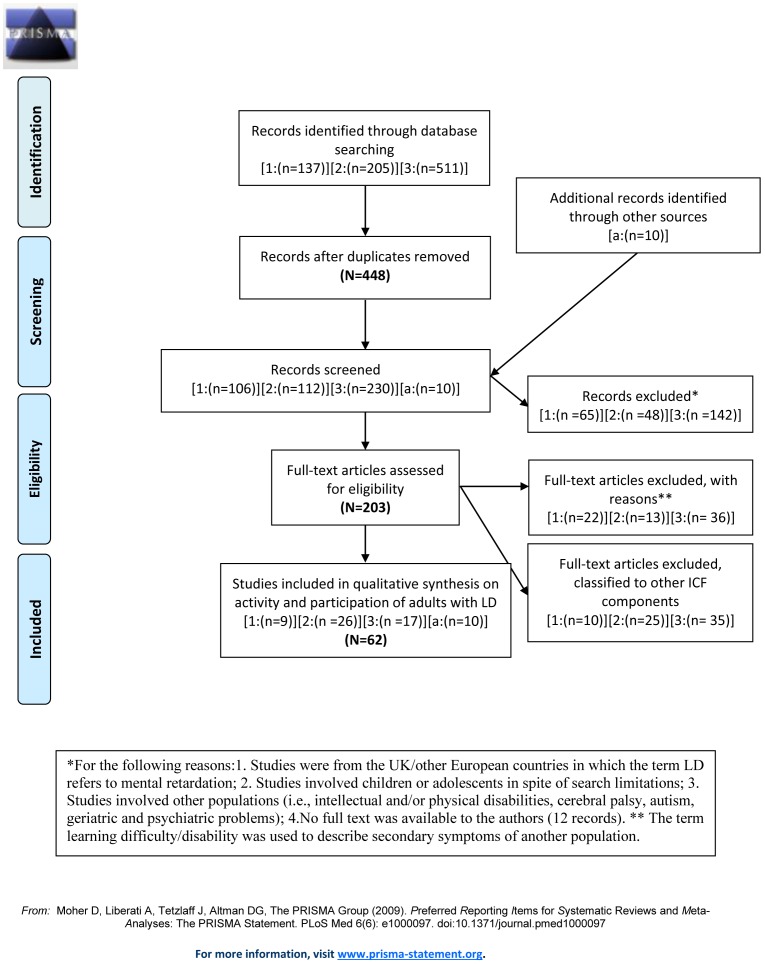
PRISMA 2009 Flow Diagram describing the three stages search process conducted for this literature review.

This systematic literature review was carried out according to the guidelines of Preferred Reporting Items for Systematic Reviews and Meta-Analyses (PRISMA) (see Table S1). No protocol exists for this review. The study selection process is summarized in Figure 1. The literature was searched in “PsychInfo”, “Eric” and “PubMed” including all of the available literature up until December 1, 2013. The search was conducted in three stages because it was difficult to obtain relevant results for *adults with LD* as a *primary health condition*. There were no limitations on publication years or types of article. However, only titles and abstracts were searched for keywords, and only peer-reviewed journal articles were included. The results were limited to English and to the population aged eighteen and over.

### Study inclusion criteria

The following selection criteria were used for the inclusion of studies: (a) the article was written in English; (b) the article related to adults; (c) LD were referred to as the primary health condition of the study population; (d) The main theme covered by the article was related, according to the authors' view, to one of the domains of activity and participation as described in the ICF model.

### Search procedure

In the first stage, the keywords ‘learning disabilities’ were combined with the keyword “activity” and then with the keyword “participation” based on the ICF core concepts. Most articles were not relevant for adults with LD, but to a variety of other diagnoses such as physical, intellectual and mental disabilities including mental retardation, autism, cerebral palsy, schizophrenia and other disorders. This stage revealed that the keywords ‘learning disabilities’ refer to mental retardation in the UK and some other European countries. In addition some of the results related to children and adolescents in spite of the age limitation.

The keywords for the second stage of the search were chosen according to the common keywords found in use at the first stage of the search. In the second stage the keywords ‘learning disabilities’ were combined with the keyword ‘adults’ and with each of the following keywords: ‘employment’; ‘social skills’; ‘daily performance’; ‘leisure’. In addition, a NOT UK limitation was added to the searches.

However, the second stage of the search also yielded few relevant results. Therefore, in the third stage of the search, ICF terminology was used in order to achieve better coverage of other domains of “activity and participation”. In this stage the keywords ‘learning disabilities’ were combined with the keyword ‘adults’ and with each of the following keywords: ‘knowledge’; ‘tasks’; ‘communication’; ‘mobility’; ‘self-care’; ‘domestic’; ‘interactions’; ‘economic’; ‘community’. In addition, the NOT UK limitation was added to the searches again.

In total, 853 records were identified through the entire search process and only 132 articles (15.47%) were relevant for adults with LD as a primary health condition. These articles included the 10 additional relevant records which were identified through other sources. It is important to note that in spite of the addition of the keyword ‘adults’ and the limitation of NOT UK all stages of this review resulted in irrelevant articles. Throughout the search process, articles from the UK and other countries in which the term LD refers to mental retardation or other major cognitive impairments as well as articles from other places where LD was presented as a secondary diagnosis and titles which dealt with children or adolescents were duly removed.

### Classification according to the ICF components and domains

A procedure of classification according to the ICF components/domains was conducted after each phase of the search. It appeared that not all articles included in this review could indeed be classified into the ICF component of “activity and participation” or according to the keywords which led to finding them. Therefore, each article was classified firstly according to the main topic being covered into one of the ICF components (“health condition”, “body functions and structures”, “contextual factors” or “activity and participation”).

After this classification only 62 articles out of the 132 articles which related to adults with LD remained classified as related to one of the activity and participation domains (see Table 1 for an overview of these articles). These articles were published between 1981 and 2012. It is important to note that in cases when an article was considered by the authors as relevant to more than one of the ICF components or domains, it was classified according to the main theme it covered as viewed by the authors. Findings of the 62 articles which were found in this review as related to adults with LD and classified into the domains of activity and participation are described below.

**Table 1:** Classification of 62 articles to domains of activity and participation according to the ICF model.

Domains of activity and participation	No. of Articles	List of Articles (By reference number # and authors)
Major Life Areas	32	{Adelman and Vogel, 1993 #20}; {Cameto, Knokey, and Sanford, 2011 #21}; {Chapman, Tunmer, and Allen, 2003 #22}; {Dickinson and Verbeek, 2002 #23}; {Doren, Lindstrom, Zane, and Johnson, 2007 #24}; {Duquette and Fullarton, 2009 #25}; {Fourqurean et al., 1991 #26}; {Freeman, Stoch, Chan, and Hutchinson, 2004 #27}; {Gerber and Price, 2003 #28}; {Gerber et al., 2004 #29}; {Gerber and Price, 2008 #30}; {Goldstein et al., 1998 #31}; {Goodman, 1987 #32}; {Greenbaum et al., 1996 #33}; {Haring and Lovett, 1990 #34}; {Hoffmann et al., 1987 #35}; {Levine and Edgar, 1995 #36}; {Madaus, 2006 #37}; {Madaus, Gerber, and Price, 2008 #38}; {Madaus, Zhao, and Ruban, 2008 #39}; {Mathis and Roessler, 2010 #40}; {McDonald, 2009 #41}; {Miller et al., 1991 #42}; {Miller, Snider, and Rzonca, 1990 #43}; {Murray et al., 2000 #44}; {Raskind et al., 1999 #45}; {Reis, McGuire, and Neu, 2000 #46}; {Scanlon and Mellard, 2002 #47}; {Shapiro and Lentz, 1991 #48}; {Siegel and Gaylord-Ross, 1991 #49}; {White, 1992 #50}; {Young et al., 2002 #51}
General Tasks and Demands	12	{Gerber, 2012 #3}; {Fafard and Haubrich, 1981 #52}; {Haring, Lovett, and Smith, 1990 #53}; {Luftig and Muthert, 2005 #54}; {Malcolm et al., 1990 #55}; {Mellard and Hazel, 1992 #56}; {Posthill and Roffman, 1991 #57}; {Shessel and Reiff, 1999 #58}; {Sitlington, Frank, and Carson, 1993 #59}; {Sitlington, 1996 #60}; {Weller, 1994 #61}; {Werner, 1993 #62}
Learning and Applying Knowledge	12	{Bahl, Plante, & Gerken, 2009 #63}; {Butler, 1995 #64}; {Coleman, Gregg, McLain, Bellair, 2009 #65}; {Gerber, Schnieders, Paradise, Reiff, Ginsberg, and Popp, 1990 #66}; {Gregg, Bandalos, Coleman, Davis, Robinson, and Blake, 2008 #67}; {Grunow et al, 2006 #68}; {Isaki, Spaulding, and Plante, 2008 #69}; {Magajna et al., 2003 #70}; {McCue, Goldstein, Shelly, and Katz, 1986 #71}; {Mellard and Patterson, 2008 #72}; {Richardson, Harris, Plante, and Gerken, 2006 #73}; {Shafrir and Siegel, 1994 #74}
Community, Social and Civic Life	3	{Einat and Einat, 2008 #75}; {Patterson et al., 2012 #76}; {Seo et al., 2008 #77}
Communication	2	{Clement-Heist et al., 1992 #78); {Klein et al., 1988 #79}
Interpersonal Interactions and Relationships	1	{Heiman, 2006 #80}
Mobility	0	
Self-care	0	
Domestic life	0	
**Total**	**62**	

## Results

### Activity and participation of adults with LD

The classification and division of the 62 articles into the different domains of activity and participation is presented in Table 1 and will be described below. The percentages reflect the proportional part of each domain in the total of 62 articles (related to as the 100%).

#### Major life areas

The largest group of articles was classified into this domain and included 32 articles [20]–[51] (51.61%) which related to educational life, work and employment and economic life. In this review, however, these articles will be described as one group on a continuum between the poles of more and less successful adaptation in these life areas.

Exemplary positive results in the “major life areas” domain demonstrated that most adults with LD who participated in a 4-year college program experienced high levels of employment satisfaction (e.g. [33]) and employment self-efficacy (e.g. [39]). Furthermore, graduates with LD achieved levels of full-time employment, benefits, and salaries that were competitive with statistics of the general American workforce population (e.g. [37]). The same picture was presented in the U.S. and Canada [29].

Some important issues are noteworthy with regard to these “successful” adults with LD: (a) implications of the LD on their work (i.e., difficulties with reading, writing, mathematics, memory) were present in spite of the general success (e.g. [33]); (b) they developed compensatory strategies in order to overcome their difficulties (e.g. [37]); (c) later on they recalled more difficult, less enjoyable educational experiences [32]; (d) a high percentage of the adults with LD who were integrated in competitive and satisfying employment still avoided disclosing their disabilities in their workplaces (e.g. [29]) due to concerns about negative impacts on their relationships with supervisors or co-workers (e.g. [37]).

Less optimistic results in the “major life areas” domain indicated that young adults with LD were significantly less likely to attend 4-year college programs or to graduate from these programs if attending (e.g. [44]) and that significant differences existed between people with LD and their non-disabled peers in favor of the latter with regard to post-college employment outcomes (e.g. [36]). Within a few years in the workforce, the incomes of adults without disabilities exceeded those of adults with LD [31]. Furthermore, in a 20-year follow-up study it was demonstrated that the socio-economic-status of adults with LD was much lower than that of their parents [45].

The findings related to adults with LD who did not attend colleges appeared to be highly variable. The authors explained such educational and work employment outcomes by different personal and contextual factors. For example, it was found that personal factors (e.g., financial autonomy, peer influence, gender) and environmental community factors (e.g., use of community resources, community mobility) differed among groups of young adults with LD who participated in alternative postsecondary education experiences [42]. It was mentioned that employment success was more likely among young adults with LD with high math abilities who were employed during high-school and whose parents actively participated in their education [26]. Furthermore, gender and their own belief in change were significant predictors of hourly wages of employed participants with LD, while career-related knowledge was a significant predictor of job satisfaction [40].

The last results described in the domain of “major life areas” are those of special education high-school graduates. These results are also varied and demonstrated that between 59% [34] and 90% [48] of the subjects were competitively employed.

#### General tasks and demands

12 articles [3], [52]–[62] (19.35%) were classified into this domain. These articles presented a decentralized mixture of results which related to performing daily routines, handling stress and other psychological demands of daily life for adults with LD according to the definition for this domain [17]. The main theme of these articles was the level of adaptation of adults with LD to the general tasks and demands of adult life.

A successful adaptation of adults with LD to the “general tasks and demands” of adult life was described, for example, by Werner (1993) who traced the development of 22 children with LD and 22 matched controls. Most subjects with LD made successful adaptations to adult life as expressed in data regarding their marriages, divorces, and employment rates, which were similar to the cohort as a whole [62]. Other articles described less successful adaptations of people with LD to the “general tasks and demands” of adulthood. For example: Malcolm, Polatajko and Simons (1990) found that 83.8% (N = 80) reported problems in at least one activity of daily living, mainly in daily organization, banking, time and home management [55]. In another example, Shessel and Reiff (1999) examined the life experiences of 14 adults with LD. Negative impacts of the LD included difficulties in daily living, social isolation, and damage to emotional health [58].

#### Learning and applying knowledge

12 articles [63]–[74] (19.35%) were classified in this domain. These articles focused mainly on the academic characteristics of people with LD. For example, Grunow, Spaulding, Gomez and Plante (2006) compared adults with and without LD and demonstrated that the group with LD showed poorer sensitivity to statistical information in speech input [68]. Magajna, Kavkler and Ortar-krizaj (2003) demonstrated significantly lower literacy proficiency among a group with self-reported LD [70] and Mellard and Patterson (2008) found that specific LD contributed significantly to variance in reading levels when controlling for age and IQ [72].

#### Community, social and civic life

Three articles [75]–[77] (4.83%) were classified into this domain and described extremely different results concerning adults with LD. On one pole, Seo, Abbott, and Hawkins (2008) compared adults with and without LD (N = 571) and found no significant differences between the groups at age 24 in the areas of schooling, employment, income, receipt of public aid and involvement in crime [77]. On the other pole Einat and Einat (2008) reported high rates of LD (69.6%) among a random sample of 89 Israeli inmates. In addition, significant relationships were found between LD, number of years of education and the age of the onset of criminal activity [75]. In another example, it was found that LD were overrepresented among a sample of homeless adults (N = 133) [76]. In this study LD during childhood had significant relationships with self-reported educational attainment and lifetime duration of homelessness as well as with a range of problems of mental health, physical health and substance use [76].

#### Communication

Two articles [78], [79] (3.22%) were classified into this domain. Klein, Moses, and Altman (1988) investigated responses to communication-assessment questionnaires. Results indicated that young adult students with severe LD and their vocational educators had similar perceptions of their communication performance. All rated problem solving abilities lower than verbal expression, comprehension, and social communication abilities. In addition all participants rated social communication abilities of the students with LD as lower than comprehension [79]. Clement-Heist, Siegel, and Gaylord-Ross (1992) described a study of multiple-baseline design conducted to investigate the effects of measured behaviors on four high-school seniors before and after vocational social skills training [78]. The authors concluded that their model was a powerful way to promote generalization of behavioral communicative skills, yet the small sample size limits the validity of such a conclusion.

#### Interpersonal interactions and relationships

One article (1.61%) was classified in this domain. Heiman (2006) reported a significant difference in perceived social support, in favor of students without LD (n = 191) compared to their controls (n = 190) [80].

#### Mobility; Self-care; Domestic life

No articles at all (0%) were classified into these domains. However, with regard to “mobility” some data could be gathered from two articles which were classified with the domain of general tasks and demands. Haring, Lovett, and Smith (1990) found that 69% of a random sample of students with LD (n = 64) had driver's licenses and cars [53], while Luftig and Muthert (2005) found even higher rates of 94% in a small sample of 17 subjects with LD [54]. Regarding “domestic life”, Haring et al. (1990) found that 79% out of the randomly selected sample of graduates with LD (n = 64) with a mean age of 21 years, reside with their parents or other relatives. The authors suggested that the subjects' involvement and/or independence in domestic life activities was still limited [53].

## Discussion

The aim of this review was to survey the literature on the *activity* and *participation* of adults with LD based on the International Classification of Functioning, Disability and Health (ICF) concepts. Through this procedure of reviewing literature, it was found that the search for articles on *activity and participation* of adults with LD generally resulted in a small percent of actual relevant results to the population of adults with LD as a primary health condition. The difficulty in finding relevant literature may be attributed not only to the use of ICF terms, but also to the ongoing discussion of the essence of LD which includes its definitions (e.g. [2]), debated identification methods (e.g. [81], [82]) and differences in research samples (e.g. [83]). Furthermore, it has been recognized that current literature on adults with LD is limited (e.g. [3], [77]). The difficulty in defining LD is intensified in practice due to the complexity of the issue of LD as described in the beginning of this review. Concurring associated disorders and health conditions which affect the daily *activity and participation* of adults with LD lead to the involvement of different professionals in the planning of intervention programs for the population with LD and their application. The large variability in life outcomes of adults with LD as described in this review matches what is known about the complexity of the issue of LD.

In the current literature review, most of the articles that were classified in the component of *activity and participation* related to *learning and applying knowledge* and to the *major life areas* of education and employment. However, identification of relevant literature to other activity and participation domains was a difficult task even though some articles which examined *activity and participation* among adults in mixed life areas demonstrated that deficiencies in *activity and participation* indeed exist in various areas in the lives of adults with LD (e.g. [55], [35], [84]). Yet the limited literature revealed only hints about what happens to this population and further extensive investigation is still required.

As described in the beginning of this review, the common definitions and identification methods of LD are based on specific difficulties of people with LD in academic studies (e.g. [6]). It is possible, therefore, that due to the focus of the definitions on the academic deficiencies of this population, research and practice have been directed over the years to the characteristics of *learning and applying knowledge* and to the *major life area*s of academic studies, to employment and economic outcomes, while other important life domains were left out of the spotlight of research and practice among adults with LD.

According to Gerber (2012) the main reason it is difficult to investigate the variety of life outcomes of people with LD through adulthood is that the field has not yet adopted a conceptual model to investigate these issues [3]. In light of the findings of this review, it is therefore suggested that the ICF terminology could be used to describe adults with LD in a manner that may generate a common language among the various professions and help researchers and clinicians working with this population to recognize that an examination of the whole picture is required in order to understand it fully. A better understanding of daily activity and participation of adults with LD may contribute to better-planned programs, thus improving their general activity and participation and perceived quality of life.

### Limitations and future research directions

This review has limitations which can direct future studies. Search words derived directly from the new concepts of the ICF model may not have led to the required results (e.g. [85]). Selecting more common search words, as was done in the second phase of the search, may have resulted in more relevant articles for this review. In addition, the search words in the second phase of the search were derived from the results of the first phase and may not have encompassed all of the domains of activity and participation in the ICF model. Therefore it is suggested that a comprehensive preliminary study to identify additional relevant search words may lead to some further relevant results for other domains of activity and participation.

### Conclusions and Implications

This article described a review of the literature on the activity and participation of adults with LD while using ICF concepts. Out of 853 records which were identified, 458 records were screened. Only 135 records were relevant for adults with LD as a primary health condition out of which 62 related to *activity and participation* of adults with LD.

This literature review demonstrated that adults with LD may experience limitations and restrictions in activity and participation in domains of communication, interpersonal interactions and community, social and civic life. However, much of the literature focused on their activity and participation in the life areas of education and employment. Therefore this review proposed that the focus of the LD definitions on academic deficiencies may have led research and practice to concentrate on these areas, while leaving other life domains such as mobility, self-care and domestic life almost completely untouched.

It was suggested that a use of the ICF activity and participation perspective may help to establish a common language and a broad understanding of the population of adults with LD among the different professionals who are involved in the evaluation and intervention programming of the LD population in order to better address their needs. Furthermore, this perspective may imply that a larger picture still needs to be revealed.

## Supporting Information

Figure S1 **Components of the ICF model (WHO, 2001).**
(TIF)

Table S1 **PRISMA 2009 Checklist.**
(DOC)
