# Supplementary figures and images for: Activity and Participation Characteristics of Adults with Learning Disabilities - A Systematic Review
Source: PLoS One. 2014 Sep 3;9(9):e106657. doi: 10.1371/journal.pone.0106657 (PMC4153678; doi:10.1371/journal.pone.0106657)

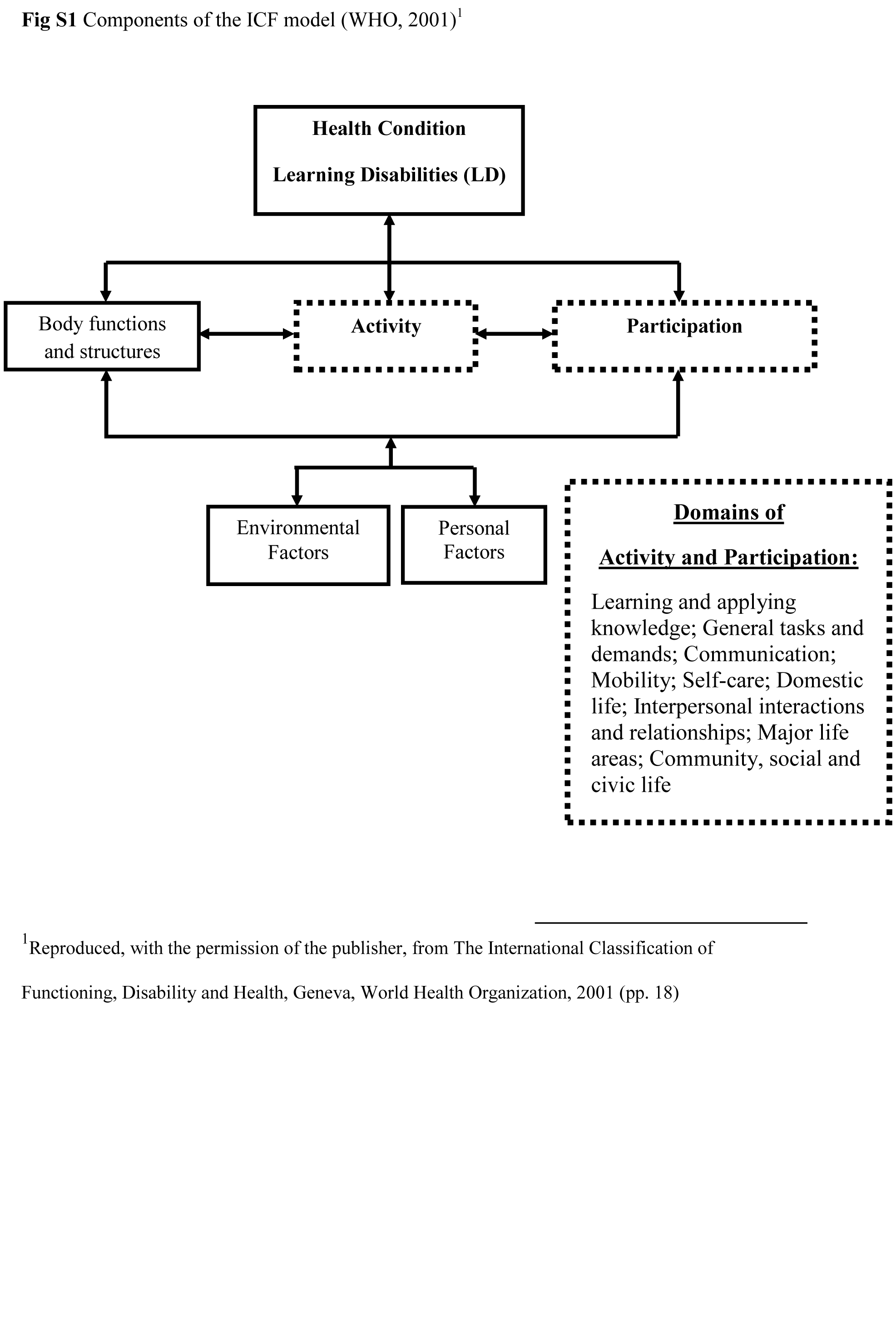

Supplement: Figure S1 — Components of the ICF model (WHO, 2001). (TIF) [file pone.0106657.s001.tif]
